# First District Dental Society of New York

**Published:** 1886-03

**Authors:** 


### FIRST DISTRICT DENTAL SOCIETY OF NEW YORK.

Reported Expressly for the Independent Practitioner.

This society held its regular monthly session at the depot of the S. S. White Dental Mfg. Co., on the evening of February 2d; the President, Dr. W. Carr, in the chair. Dr. C. F. W. Bodecker, chairman of the Clinic Committee, reported as follows:

There were over one hundred persons present at the clinic to-day, and the operations performed were very instructive. Dr. C. B. Parker, of Brooklyn, presented a patient about fifteen years of age who, three years ago, broke the right upper central tooth across the middle in a horizontal direction, exposing the pulp, which at that time was capped, and the tooth filled with Smith's oxy-phosphate of zinc. Last fall Dr. Parker removed the cement, and restored the contour of the tooth by a piece of porcelain. The anchorage for the latter was obtained in the dentine near the boundary of the enamel, around the pulp, which was found alive. The color of the porcelain had been matched so perfectly, and the adjustment to the tooth had been done so skillfully, that close examination was required to detect the former defect. Dr. Parker, in the clinic this afternoon, adjusted a similar piece of porcelain upon a left upper central tooth, but this case was not as favorable as the one mentioned before. The tooth had lost its mesial corner, and the adjacent enamel was very thin and transparent, making it difficult to match the porcelain, but the operation, when completed, was very neatly done.

Dr. C. H. Land, of Detroit, Mich., exhibited his new muffle gas furnace, which, by all experts present, was acknowledged to be the best gas furnace ever presented to the dental profession. In this furnace, the construction of which is based upon scientific principles, the gasing of the work is entirely prevented. Dr. Land also presented his new porcelain-faced crown and bridge work, which is made as follows: A piece of thin platinum (about No. 30) is tightly fitted around the broken crown or root of a tooth, which has been previously trimmed on its labial (or buccal) and proximate surfaces sufficiently to make room for the new material to be adjusted; then an ordinary plain plate tooth, of required shape and shade, is ground out so thin that, when held in place upon the platinum cap, it occupies the proper position. This thin porcelain face is then baked upon the platinum cap with a body especially prepared for the purpose.*

Dr. C. A. Caulkins exhibited in his own mouth an extensive piece of bridge work, made by the Sheffield Co., which had utterly given out with three months' wear. Dr. Caulkins, being present at the meeting, made the society a present of the bridge work.

Dr. Wm. T. La Roche exhibited the models of a complicated case of irregularity, involving both the upper and lower jaws.

Dr. J. B. Lawrence exhibited the new Diehl electric motor, and a new cement, the powder of which is composed of the oxides of zinc, tin and aluminum, and the fluid is a solution of phosphoric acid.

Dr. Oliver exhibited the Cogswell disk carrier and guard.

A gentleman distributed some samples of a new disinfectant, called "Omico," which may be used in cases to which the well-known Listerine is applicable.

Dr. J. L. Williams exhibited a new form of plain tooth, applicable for single crowns, as well as bridge and plate work.

Dr. C. F. W. Bodecker presented a lady with a swelling of the left side of the lower jaw. This enlargement was not constant. It occupied a position under the roots of the third molar, which, some years ago, was extracted. The first and second molars were filled with oxyphosphate of zinc, and their pulps apparently in a healthy condition; and, as Dr. B. was unable to find the seat of the trouble, Dr. L. Waldstein, a physician, was consulted, who pronounced it to be caused by a disturbance in the circulation, brought about by a displacement and chronic inflammation of the uterus. Dr. Wm. H. Atkinson, who saw the patient this afternoon, agreed with the diagnosis, and added that in the place where the third molar had been extracted the blood vessels had become varicosed. The treatment advised was manipulation and applications of iodine.

In this patient's mouth there was also present a very large gold filling, occupying the whole of the lingual cusp, from the gum downward, of the left upper first bicuspid, which had been done by Dr. Bodecker, principally by the Herbst method. The filling was pronounced by those who examined it, Drs. Wm. H. Atkinson and E. P. Brown included, to be perfect in every respect.

Dr. C. F. W. Bodecker showed a piece of steel tape measure, upon which the figures and the lines dividing the inches and their fractions are somewhat elevated. This steel tape measure, which

---

*Dr. Land is writing an article on this subject, which will be published in the INDEPENDENT PRACTITIONER.

was very thin, was highly recommended for burnishing the edges of proximate gold fillings, drawing it, in polishing, from one side to the other like a piece of emery paper.

The Secretary, Dr. Nash, presented to the society a plaster model of a large osseous tumor sent to the Society by Dr. Genese, of Baltimore; also a brief paper giving a description of the case, and its treatment. A man about twenty-seven years of age entered the Baltimore Hospital with a large swelling on the right side of his face, the result of a blow from the handle of a windlass, some thirteen years before. He was in good health, and the swelling gave no pain, yet it was unsightly, and he wished it removed. Dr. Genese was sent for by Dr. Billings, the surgeon in charge, for consultation, and the tumor was diagnosed non-malignant. It extended from the external plate of the superior maxilla, involving the malar and zygomatic portions. It was deemed unsafe to administer an anæsthetic, for fear of hemorrhage and possible strangulation from flow of blood. To avoid a scar it was decided to operate wholly within the mouth. The cheek was distended, the periosteum dissected from the tumor and held away with forceps. An incision was made along the border of the tumor, and this followed with an inverted-cone bur in the dental engine, to make a channel. Then with knife edge and chain saw the entire growth was removed in three sections. The rough edges were burred off and its surface covered with the periosteum. The roots of the teeth were left with a thin covering of bone, the mouth cleaned and the lesion dressed with tannin, glycerine and iodoform. "Nervine Vita" was applied before and during the operation to prevent pain and check the hemorrhage, which proved successful for each purpose, although a five per cent solution of the oleate of cocaine was tried without effect. The patient made a good recovery, showing only a slight enlargement of the zygomatic aspect of the arch.

Under incidents of office practice, Dr. W. H. Dwinelle exhibited several casts representing cases of enamel erosion. From his success in treating these cases he would say a word of encouragement to others who might have to deal with similar ones. Such cases seem at times to come in groups; to pour in upon us. Dr. Darby's recent paper left us at sea regarding the causes and the cure of erosion; but here were cases fully diagnosed, with remedies pronounced. One cast represented teeth in the mouth of a lady, showing much erosion, the decalcification extending nearly through to the dentine. He polished the surfaces smooth, and although three years have since passed, the teeth then treated have remained perfect. Dr. Dwinelle alluded to experiments made by the late Dr. Westcott, of Syracuse, who subjected teeth to various tests with very interesting results. Dr. Westcott found that acids of an astringent nature, as from some fruits, were ready solvents of enamel. Dr. Dwinelle referred to a lady with incisors exceedingly sensitive and badly eroded, occasioned by partaking freely of grapes. He cut away and polished the rough surfaces, and in some places even entirely through the enamel, and so arrested the trouble.

Dr. C. E. Francis reported a case where the superior central incisors were much eroded. They had become so within six months, and their roughened condition was due to eating bountifully of grapes and grape fruit. She had been in the habit of pressing the fruit against her teeth to extract the juices and pulps. Dr. Francis also mentioned a case of serious and extensive erosion in the mouth of a young lady who had recently recovered from typhoid fever. Her teeth all around, which a few months before seemed almost perfect, were now very rough and pitted. He had decided to polish their surfaces in the manner indicated by Dr. Dwinelle. He mentioned another case where, many years ago, a lad was brought to him whose teeth were riddled with enamel pits and serrations, the result of scarlet fever during childhood. As the engine was not then in use, he trimmed the surfaces with chisels and files, then polished with points of stone. This secured their preservation.

Dr. Francis spoke of a gentleman who came to him two-and-a-half years ago, whose teeth were coated with dark stains and loaded with festoons of salivary calculus. His gums were purple, swollen and ready to bleed at the slightest touch, and the teeth so sore as to be of no use whatever for masticating. The extraneous collections were removed, the teeth polished and the gums treated until they presented a healthy appearance, and the teeth restored to usefulness. The patient was admonished that if he again neglected his teeth the same trouble would reappear and he might lose them. Last summer (nearly two years after) he again presented himself for treatment. The mouth was in a worse condition even than at his first visit, but was again put in as good order as possible. Yesterday brought the patient once more, and the appearance of his teeth and gums worse than ever. The salivary calculus had collected in such masses as almost to suggest the use of a plow to work it off. The inferior incisors were so loose that they could be removed with the fingers.

Another case was that of a lady who was warned by Dr. Francis, some eighteen years ago, that if she did not take better care of her teeth she would certainly lose them. They were loaded with accumulations and her gums much inflamed. She informed him that her former dentist (who had since died) had given her a similar warning some time before, and predicted that in ten years she would be toothless. Dr. Francis did all in his power, both by personal effort and by advising a plan for her to persue, to aid her in efforts to preserve her teeth. She called at intervals, but each time found her gums as bad as before, and finally several of the teeth became so loose as to fall out. A few of them decayed, but the patient would not have them filled. A few days since one of these gave her much pain, and she applied to a dentist near at hand for relief. This fellow, on looking into her mouth, at once declared that her teeth had been allowed to go to ruin through the neglect of her dentist! Furthermore, he told her that *he* could have saved her teeth, and could do so yet. He made an application to the aching tooth, but without affording the slightest relief, so the lady went to Dr. Hasbrouks, took gas and had it extracted. Dr. Francis brought the tooth to the meeting, and it was passed around the room for examination. It was badly decayed; indeed, the dentine was nearly all gone, and the enamel half destroyed by decalcification. Calcareous deposits also had worked their way over the surface of the root, extending on one side quite to the apex. Dr. Francis desired to know if other dentists had similar experiences, and if any one believed that a tooth in the condition of the one presented could be saved.

Dr. J. L. Williams, of Philadelphia, presented new systems of crown and bridgework, inventions of his own, and apparently of much merit. Dr. Williams referred to the annoyance and injury incident to wearing partial dentures, especially where but a single tooth was wanted. He stated that a few in the profession had avoided the use of a plate in some cases where bicuspids and molars had become lost, by soldering a backing to an ordinary plate tooth and allowing projections to extend into cavities of the adjoining teeth, and secured by fillings. But although in some instances these seemed to give a degree of satisfaction, they could hardly withstand the severe strain caused by the ordinary process of mastication. Dr. Williams' newly designed teeth contain four strong platinum pins, two on each end, embedded well into the body of the tooth, and so diverging at the point where they enter as to give the greatest possible strength. These projecting pins may be bent in any direction to conform to cavities in adjoining teeth, or may be soldered to form a loop.

Dr. Williams called attention to a second device where the same principle is applied to incisors. He also referred to yet another device, which he feels sure will revolutionize the present methods of constructing bridge-work, and obviate the objections to this style of work, as at present constructed. The teeth have no pins, but a groove extends across the longest diameter on two sides of each. They are so arranged that the backing will not show from the front. A backing of platinum is fitted to each groove, also a little staple attached to it and soldered to the bridge. Teeth thus made are stronger than those new in use, and less likely to give ont. If, however, they happen to break, new ones of the same pattern can easily be fastened to the bridge by cement of oxy-phosphate of zinc. Cases where these teeth are used present a better appearance, and a saving of time and expense in their construction. Dr. Williams claims that the system first presented is original in every feature, and is no infringement on the rights or claims of any one. He therefore presented the model, with all the rights and privileges pertaining to the use of such work, to the First District Society of New York, and through this society to the profession.

Upon motion of Dr. Bodecker, the thanks of the society were tendered to Dr. Williams in a most enthusiastic manner.

Prof. Frank Abbott read a paper on "Pulpless Teeth."

He believed that no operations in the practice of our specialty were attended with greater anxiety, or required a more thorough knowledge of remedial agents than in the treatment of pulpless teeth; but as every dentist has his own particular ideas and methods when treating such teeth, he would simply give his individual views concerning them, and state his methods of dealing with them. Dividing his subject into four sections, he proposed to consider them in order. First, he would speak of the treatment of pulpless deciduous teeth, which he asserted were always attended to with more or less uncertainty, and this in great part was due to the fact that by the time the decay extended to their pulps the roots had become absorbed, and their jagged edges were a source of constant irritation. The first step in treatment of these (and all pulpless teeth) is to thoroughly cleanse them from all the debris of the dead pulp, using warm salt water with alcohol, a weak solution of carbolic acid in water, listerine diluted with water, or any other non-irritant of a similar nature Where a deodorizer is required he suggested permanganate of potash, three grains to one ounce of water. After this he would force a spray of some germ-destroying antiseptic into the cavity. He then drills a small opening through the side of the tooth, to enter beneath the edge of the gum and extend to the pulp chamber, for a permanent vent. A thin platinum cap is fitted to the bottom of the cavity, and over this a filling is placed. The opening drilled through should be kept clear. Dr. Abbott would not attempt to fill root canals of deciduous teeth, fearing that such fillings would keep up constant irritation to the surrounding tissue. The opening from the pulp-chamber gives exit to any possible exudation, and also affords an opportunity for cleansing or medicating if needed. Where such treatment is followed, swollen face will be avoided.

Next in the order considered were pulpless permanent teeth, and these also should be well cleansed. Some of the roots of these teeth are so tortuous and contracted as to render the entire removal of dead pulps an impossibility. The successful treatment of cases of this sort depends much on the condition, whether septic or antiseptic, of whatever portions of the pulp there may be left. Dr. Abbott warned dentists against using drills for enlarging pulp canals, as they are apt to pierce through to the alveolus, and he exhibited several specimens, showing how such mischief had been done. After cleansing as thoroughly as conditions permit, he would use the same remedial agents as for deciduous teeth, or a $\frac{1}{2000}$ solution of bi-chloride of mercury. Then a pellet of medicated cotton is packed into the cavity and sealed with gutta-percha and wax. This is removed in two or three days, and the spraying and antiseptic repeated, and kept up at like intervals for about a fortnight. If no trouble has then occurred he would fill as follows: A small bit of cotton is carried to the end of the root and packed lightly, and covered with a soft paste of oxy-chloride of zinc. This is done where roots are entirely pulpless, but where any portion of a pulp remains, a small pellet of cotton is saturated with a mixture of oxy-chloride of zinc and placed loosely in the root. This is covered with gutta-percha gently pressed against it, which forces the zinc paste into every part. If pain is thus caused, the pressure must be stopped at once and the pain allowed to subside. When comfortable, the gutta-percha is removed and a filling introduced to complete the operation.

In cases where the pulp is living in one or more roots, it should be carefully treated and capped, but if any are dead they should be treated as already described.

Where an abscess is discharging through a fistulous opening in the gum, the tooth should be cleansed and a solution of chlor. zinc, 40 grains to 1 oz. water, forced into the root canals until it works through the fistula; then the canals are filled with oxy-chlor. of zinc. This can be done at one sitting, because the anti-septics have had their effect and the chloride of zinc readily destroys the pyogenic membrane which forms the pus sack. Granulation will follow, and the irritation that any subsequent operation might cause would be avoided. After operations on dead teeth the gums around them should be painted with a mixture of equal parts of tinct. aconite rad. and tinct iodine, except in cases of abscess, to prevent or relieve pericementitis.

If the tooth and roots are filled and the pain and swelling cannot be relieved by such applications, a hot raisin can be applied. A plump raisin split open, and the seeds removed, should be dipped in hot water and placed fruit side against the gum. This can be repeated every half hour for several hours, and one left there over night. The relief this gives is, in some instances, beyond comprehension.

*Dr. Francis*—Complimented Dr. Abbott's paper as embodying good practical sound sense. Referring to the application of heated raisins, Dr. Francis stated that within a few days he was sent for to see a lady with a painfully swelled cheek, caused by a devitalized inferior molar. The tooth contained a large contour filling, and the patient informed him that the roots were also filled. The gum was also much swollen, and the tooth too sore to bear the slightest touch. He advised cold applications to be kept on her cheek, and half of a roasted raisin to be placed against the gum. This at intervals was changed for fresh ones, and a saline cathartic administered. The inflammation soon became reduced, and a slight discharge of pus appeared through the gum. In two days the patient was well as before.

*Dr. F. Y. Clark*—Said that one important point was omitted in Dr. Abbott's paper, viz., the difficulty of treating with success cases where there was no fistulous opening. In treating root canals there is danger of forcing more or less debris through the foramina. He thinks it unsafe to put nerve instruments into roots. He would syringe thoroughly, inject disinfectants, alcohol or ether, and seal the cavity to remain twenty-four hours before filling. The careless use of broaches causes choking at the ends of the roots.

*Dr. Spooner*—Claims that such cases as were mentioned by Dr. Francis and others come within the province of the dentist to treat. Where teeth are covered with stains or tartar they should be cleansed. Many such troubles are due to a disordered condition of the stomach, and the abuse of this organ is the cause of other ills and defects. Food fermentations are productive of great injury to the teeth.

*Dr. Weld*—Considered the paper read by Dr. Abbott able and comprehensive. Of all subjects connected with our specialty none are more important than those referred to. Many devitalized teeth, if well treated, are tolerated in the mouth, and are useful for years, but although some may give little or no seeming trouble, cases of neuralgia may at any time be expected where dead teeth are present. He spoke of a call from a lady suffering pain produced by a superior molar, which, a year previously, had been treated by an able dentist. Getting little or no benefit, she called upon another dentist with no better result. Then she called upon the speaker, who treated and filled it carefully, but it gave her further trouble, and she called on another dentist and had it extracted. Dr. Weld referred also to another case, where a troublesome lateral incisor was removed, and upon examination the root canal was found to contain half dead pulp and half filling. He concluded by saying that in the treatment of devitalized teeth we have sometimes our successes, and sometimes our failures.

*Dr. Atkinson*—Made some remarks not very complimentary to the paper presented, nor to those who had discussed it. He, however, commended Dr. Spooner for asserting that the stomach had much to do in causing tooth trouble. Dr. Atkinson thinks we follow too much the advice of our patients, and, therefore, do not treat them as we ought. Many individuals have dead teeth that give no trouble. We can learn much by the mistakes of ourselves and others.

